# Is color an integral part of a rich mental simulation?

**DOI:** 10.3758/s13421-017-0708-1

**Published:** 2017-04-24

**Authors:** Lara N. Hoeben Mannaert, Katinka Dijkstra, Rolf A. Zwaan

**Affiliations:** Erasmus University Rotterdam, Burgemeester Oudlaan 50, Mandeville Building, PO Box 1738, 3000 DR Rotterdam, The Netherlands

**Keywords:** Color, Language comprehension, Perception, Mental simulation

## Abstract

Research suggests that language comprehenders simulate visual features such as shape during language comprehension. In sentence-picture verification tasks, whenever pictures match the shape or orientation implied by the previous sentence, responses are faster than when the pictures mismatch implied visual aspects. However, mixed results have been demonstrated when the sentence-picture paradigm was applied to color (Connell, *Cognition, 102*(3), 476–485, 2007; Zwaan & Pecher, *PLOS ONE, 7*(12), e51382, 2012). One of the aims of the current investigation was to resolve this issue. This was accomplished by conceptually replicating the original study on color, using the same paradigm but a different stimulus set. The second goal of this study was to assess how much perceptual information is included in a mental simulation. We examined this by reducing color saturation, a manipulation that does not sacrifice object identifiability. If reduction of one aspect of color does not alter the match effect, it would suggest that not all perceptual information is relevant for a mental simulation. Our results did not support this: We found a match advantage when objects were shown at normal levels of saturation, but this match advantage disappeared when saturation was reduced, yet still aided in object recognition compared to when color was entirely removed. Taken together, these results clearly show a strong match effect for color, and the perceptual richness of mental simulations during language comprehension.

Many empirical studies have supported theories of grounded cognition, which suggest that we use the same sensorimotor regions in the brain during activity as during cognitive processes, through the use of mental simulations (Barsalou, 1999, 2008). It has been argued that activation of perceptual areas in the brain during language comprehension are not merely epiphenomenal but that language can, in addition to communication, serve as a control mechanism to shape mental content (Lupyan & Bergen, 2015). One such experiment examined whether we create mental simulations of an object’s orientation when the orientation is implied in the sentence (Stanfield & Zwaan, 2001; Zwaan & Pecher, 2012). The study showed that when the implied orientation matches the orientation of the object shown in an object-verification task, that reaction times are shorter than when they mismatch, suggesting that we create mental simulations during sentence comprehension. This match advantage has also been found for visual aspects such as shape (Zwaan, Stanfield, & Yaxley, 2002), visibility (Yaxley & Zwaan, 2006), and motion (Zwaan, Madden, Yaxley, & Aveyard, 2004); has been found for children (Engelen, Bouwmeester, de Bruin, & Zwaan, 2011) as well as for the elderly population (Dijkstra, Yaxley, Madden, & Zwaan, 2004); that spoken words also rapidly activate visual representations that affect our ability to recognize objects (Ostarek & Huettig, 2017); and the shape of an object becomes activated during encoding, and not simply during retrieval (Zeng, Zheng, & Mo, 2016).

However, mixed results have been found when this sentence-picture paradigm was applied to color. For instance, Connell’s (2007) study illustrated an advantage in the mismatch condition. Connell (2007) suggested that color may be represented differently than other visual features because it is one of the few object properties that is unimodal, (i.e., it can only be perceived with the visual modality) and has been shown to be less vital to object identification than shape (Tanaka, Weiskopf, & Williams, 2001) or orientation (Harris & Dux, 2005). Thus, it should be easier for participants to ignore mismatching color information and focus on a stable object property such as shape than to ignore the matching color as it aids in solving the task demands and requires processing. Zwaan and Pecher (2012), however, conducted six replication experiments to investigate this match advantage in greater detail for object orientation, shape, and color, and found a match advantage for all three object properties. Moreover, the match advantage for color had a larger effect size than those for shape and orientation. Another study also appeared to support a match advantage for color, as reading words in a color (e.g., white ink) matching the color implied by a previous sentence (e.g., *Joe was excited to see a bear at the North Pole*) facilitated reading times (Connell & Lynott, 2009).

These contradictory findings in studies examining color as part of mental simulations prompt further questions into how we process color during language comprehension and how much sensory information we include in these simulations. One possibility is that color is an unstable visual feature in mental simulations, as the color of an object can change without eliminating the ability to recognize the object, and therefore may play a less present role in mental simulations.

One of the goals of the current investigation was to address the potential problem of color instability caused by the stimulus set used in the original study (Connell, 2007) and in the replications (Zwaan & Pecher, 2012). To address this issue, we created a stimulus set that met more stringent criteria with regard to the visual features than the earlier stimulus sets did. For example, there were some items in the previous study in which features other than color could vary (i.e., a steak that is cooked has a different shape than a steak that is raw). This problem does not occur for more carefully chosen, less variable, items, such as a red or green tomato. Therefore, in the current investigation, all potentially problematic items were removed and replaced with stimuli that could undergo a color change while their shape remained unaltered. Another difference in our stimulus set was that full-color photographs were used rather than line drawings, to allow for a more realistic representation of the described objects (Holmes & Wolff, 2011).

The second goal of the study was to examine how much sensory information is captured in a mental simulation. Color is a useful tool for exploring this, as it is the only visual feature that can be decomposed into different dimensions, namely hue, saturation, and brightness (Palmer, 1999). This decomposition is solely a color aspect manipulation as the decomposition process still allows for the object to be recognized (i.e., there is no change in shape, size, or orientation). For instance, a tomato without hue will simply become a gray tomato, maintaining its shape and preserving all other visual features. At the same time, however, changes in color, saturation or brightness affect the richness of the visual stimulus, as these dimensions alter what is typical about the visual properties of the stimulus. Thus, if these dimensions affect the richness of the visual stimulus, is it necessary to represent them in a mental simulation? When one processes a sentence implying a certain color, is information regarding the saturation of the color stored? For example, when reading about a ripe tomato, would a simulation include a bright red, or would this not be as vital to the simulation as other sensory information?

Our current study explored how much sensory information is included in mental simulations by conducting four experiments, using the same experimental paradigm as Connell (2007) and Zwaan and Pecher (2012) where sentences are used to imply a certain color, followed by an object-verification task. For example, the sentence *The driving instructor told Bob to stop at the traffic lights* is used to imply a red traffic light, rather than explicitly stating *The driving instructor told Bob to stop at the red light*. After reading a sentence implying a certain color, participants see either a matching (e.g., red light) or mismatching picture (e.g., green light) and have to press a button on the keyboard verifying whether the pictured object was mentioned in the previous sentence, where the correct answer to experimental items always required a “yes” response.

The first experiment was conducted as a conceptual replication of Connell’s (2007) and Zwaan and Pecher’s (2012) experiments on color, to resolve which of the contradicting findings has more empirical support. Given the previous literature, we predicted to find a significant match advantage. Experiment 2a and 2b addressed the question of how much perceptual information is included in a mental simulation. This was accomplished by lowering the saturation of the pictures used in Experiment 1 to the lowest level at which the hue could still be recognized. It is possible that by reducing the level of saturation in the picture there is less of an overlap with what is currently being simulated, which could lead to there being less facilitation of a response in the match condition under low levels of saturation. A further possibility is that rather than the match condition acting as a facilitatory mechanism, the match effect exists due to there being a vivid difference between what is simulated and what is pictured in the mismatching condition. Reducing the level of saturation would then reduce the disparity between the picture and the simulation, leading to faster responses in the mismatch condition. In other words, there would be less interference. Experiment 3 examined whether a match advantage still exists when objects are shown completely in grayscale. This is of interest for several reasons. First, if a match advantage does appear under low levels of saturation, then it should disappear when the pictures are shown in grayscale. Second, studies have shown that color does aid in object recognition (Bramão, Reis, Petersson, & Faísca, 2011). With this in mind, we expect that participants’ response times (RTs) in Experiments 1 and 2 will, overall, be faster than in Experiment 3, where no color is present.

## Experiment 1

### Preregistration

The predictions, exclusion criteria, design, methods, analyses, and materials of all the experiments reported in this article were preregistered in advance of data collection and analysis on an online research platform—Open Science Framework (OSF; see Nosek & Bar-Anan, 2012; Nosek, Spies, & Motyl, 2012, for a detailed discussion on replications and preregistration). This ensured that confirmatory procedures (hypotheses testing) were conducted according to a priori criteria. In the current article, a clear distinction between confirmatory and explanatory analyses was made, as suggested by De Groot (1956/2014). The post hoc analyses are included in the Exploratory Analyses section.

### Method

#### Participants

Two hundred and five participants were recruited via Amazon’s Mechanical Turk^1^ (87 males, mean age 37.78 years, range: 20–87 years). The participants were paid $1.50 for their participation.

#### Materials

The experimental flow was programmed in Qualtrics Survey Software. It allowed for an automatic collection of information such as Browser Type, Browser Version, Operating System, Screen Resolution, Flash Version, Java Support, and User Agent for each participant.

#### Pictures

Thirty-two pictures were selected as experimental items and 16 as filler items. The pictures were obtained from the internet (Google image search engine). Picture size was unified across the trials: none of the pictures exceeded 300 × 300 pixels (approximately 7.9 × 7.9 cm onscreen). The objects depicted in the images had one dominant color (e.g., green in the green traffic light picture). The experimental items formed 16 pairs of objects, and pictures within a pair differed in color (i.e., red traffic light vs. green traffic light). The pictures of the objects within a pair were matched in terms of size and shape to ensure that neither shape nor size could be a confounding variable.

#### Sentences

**Fig. 1:**
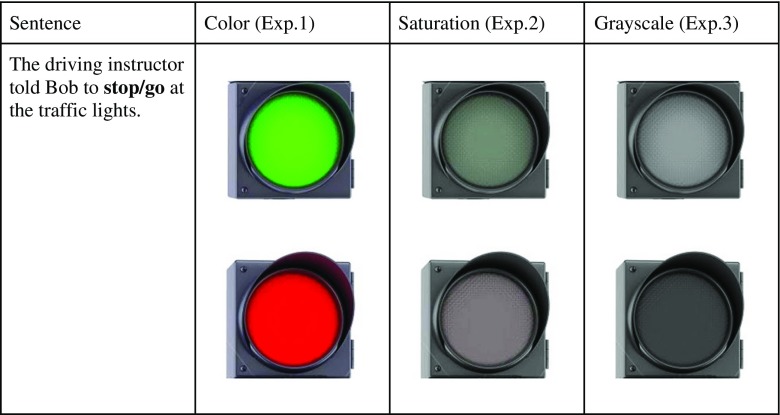
Example of stimuli material used in each experiment. A matching picture illustrates that color was implied by the sentence (i.e., red when asked to stop at a traffic light), and a mismatching color illustrates that this color was not implied by the sentence. (Color figure on line)

There were 48 sentences constructed in total: 32 experimental and 16 filler sentences. Similar to the pictures, experimental sentences also formed pairs, with one sentence implying one color of an experimental and the other implying the color of the remaining item of the pair (see Fig. 1). Participants viewed 16 experimental sentences and 16 filler sentences. Eight comprehension questions were added to half of the fillers to ensure that participants did not simply “skim” through a given sentence but read and understood it. Additionally, six sentence-picture pairs were used as practice trials.

#### Design and procedure

Design and procedure were almost identical to Connell (2007). There were four picture-sentence combinations, so four lists were created so that each group was presented with one of the possible combinations (see Fig. 1). Each list contained the same proportion of experimental and filler sentences, and the various colors present in the pictures were spread evenly across groups. Thus, the experiment was a 2 (sentence version: Type 1, Type 2) × 2 (picture type: match, mismatch) × 4 (lists) design, with sentence version and picture type as within-subjects variables and lists as a between-subjects variable.

**Fig. 2:**
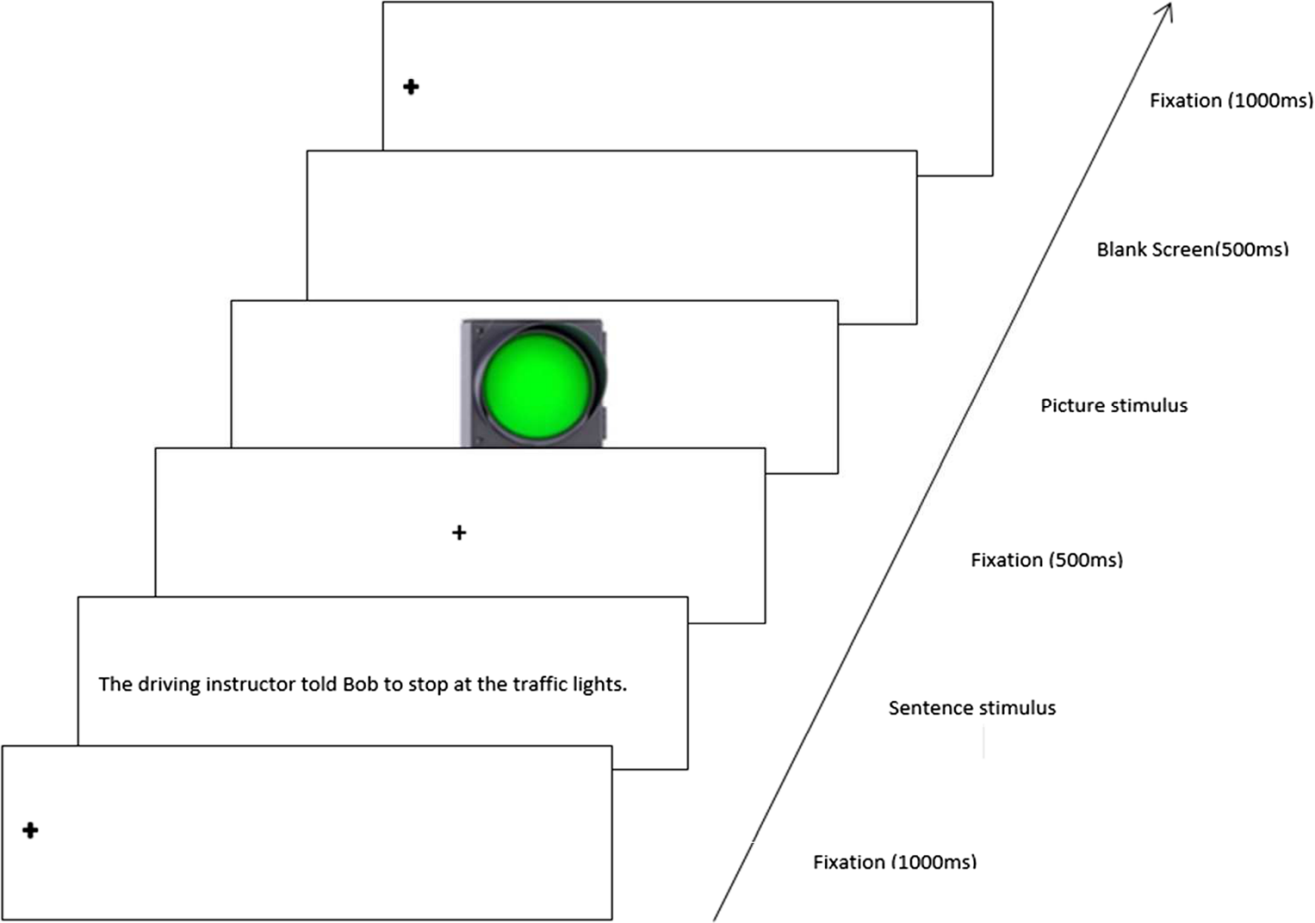
Example of an experimental trial sequence. (Color figure online)

Participants were instructed to read the sentence and press the spacebar when they had understood it. They were informed that each sentence would be followed by a picture, and their task was to decide whether the depicted object was mentioned in the preceding sentence. Participants were asked to respond as quickly and accurately as possible by pressing the *L* key for *yes* and the *A* key for a *no* answer. The responses were collected and saved automatically by the Qualtrics Survey Software. The instructions presented to the participants warned them that occasionally they would receive a question to test their comprehension of the previous sentence, to which they would either agree (by pressing the *L* key) or disagree (by pressing the *A* key). The trial sequence was as follows: a left aligned vertically centered fixation cross appeared on the screen for 1,000 ms followed by the sentence. After a spacebar press, a fixation cross was presented in the middle of the screen for 500 ms followed by a picture. When a yes/no decision was made, a blank screen appeared for 500 ms, after which another trial began (see Fig. 2).

All experimental items required a *yes* response, and all filler items required a *no* response. As participants received six practice trials, it was clear for participants when a *yes* and *no* response was required.

### Results and discussion

The data from 42 participants were discarded from further analysis: five participants were not native English speakers, six participants reversed the response keys (which was indicated by accuracy scores at or below 21%), and 31 had accuracy scores lower than 80%. The drop-out rates were not equally spread across the four lists. To make the cells equal and enable parametric tests to be run, the required number of participants who were at the bottom of each list was removed (total of 25). After the exclusion process, each list included 34 participants (136 participants in total). For the analysis, we collapsed participants across lists as list was not a factor in our preregistered plan of analysis. Finally, one item was removed from the analysis as the average item accuracy was below 80%. This would indicate that participants did not believe the pictured object belonged to the preceding sentence.

Earlier research using the picture verification paradigm has used the median instead of mean reaction times (e.g., Stanfield & Zwaan, 2001; Zwaan & Pecher, 2012; Zwaan et al., 2002). An advantage of using medians compared to means is that their use does not necessitate further decisions regarding outlier removal (e.g., whether to use cutoffs based on standard deviations, absolute RTs, or a combination thereof).

A paired-samples *t* test was conducted to investigate whether there was a match advantage for accuracy and RTs. For the RT analysis, only RTs of correct responses were included in the analysis. The participants showed significantly higher accuracy rates in the match condition (*M* = .96, *SD* = .06) than in the mismatch condition (*M* = .90, *SD* = .11), *t*(135) = 5.36, *p* < .001, *d* = 0.46, BF10 = 33380.05. The match advantage was also found in the RTs: the match condition was 104 ms faster than the mismatch condition (*M* = 1,230 ms, *SD* = 568 ms and *M* = 1,334 ms, *SD* = 676 ms, respectively). This difference was significant, *t*(135) = 3.00, *p* = .003, *d* = 0.26, BF10 = 6.88. Participants’ accuracy when responding to the comprehension questions was high (*M* = 0.79, *SD* = 0.20).

These findings support the results of Zwaan and Pecher (2012), rather than those of Connell (2007), and suggest that color, like shape and orientation, is an object property that is simulated during language comprehension.

## Experiment 2a

The results of Experiment 1 served to illustrate that sentences implying color are represented in mental simulations but makes no conclusions as to how rich these simulations are. If color is not present in mental simulations, then reducing color saturation should not affect the match advantage. If we do simulate color, however, and do so vividly, then showing a mismatching pictures in full color should lead to a larger disparity between the two conditions than when saturation of the color is reduced. Experiment 2 examined this problem by reducing color saturation to the lowest level at which the hue can still be distinguished to test whether a match effect would still appear, and whether it would be smaller than in Experiment 1.

### Norming study

A norming study was conducted in order to determine the lowest saturation level possible at which a certain hue could still be recognized using the same pictures as in Experiment 1. Twenty-four subjects were shown six different saturation levels per picture and were asked to choose the picture that had the lowest level of saturation while they could still perceive the associated hue. Picture saturation was adjusted using Microsoft Office Picture Manager’s Color Enhancement Tool (where −100 is a black and white/grayscale picture and 100 is a very intense, color-rich picture). The pictures that were selected by the majority of the participants as having the least amount of color while still being able to recognize the hue were used in the experiment.

### Method

#### Participants

Two hundred and eight participants (99 males, mean age 37.93 years, range: 22–71 years) took part in this Mechanical Turk experiment. The participants were paid $1.50 for their participation.

#### Materials

The stimuli used in the current experiment were adapted from Experiment 1, and the levels of saturation chosen for the stimuli were determined by the norming study described above (see Fig. 1). The sentences remained unchanged.

#### Design and procedure

The design and procedure of Experiment 2 were identical to that of Experiment 1.

### Results and discussion

Sixty-eight participants were excluded from the analysis (five were not native English speakers; four appeared to have reversed the keys; 14 had accuracy below 80%; and 45 participants were excluded from the bottom of the lists to achieve equal numbers of subjects per list), leading to a total of 140 participants being included in the analysis.

A paired-samples *t* test was conducted to investigate whether there was a match advantage for accuracy and RTs. The results indicated no difference in accuracy rates between the match (*M* = .96, *SD* = .07) and mismatch condition (*M* = .95, *SD* = .08), *t*(139) = 0.98, *p* = .331, *d* = 0.08, BF10 = 0.15. There was also no difference in the RTs between the match (*M* = 1,156 ms, *SD* = 558 ms) and the mismatch conditions (*M* = 1,165 ms, *SD* = 639 ms), *t*(139) = 0.25, *p* = .801, *d* = 0.02, BF10 = 0.10. Comprehension accuracy was high (*M* = 0.81, *SD* = 0.19).

## Experiment 2b

There was some concern that Experiment 2 could not be accurately tested using Mechanical Turk as there would be no way to control for the brightness of participants’ computer monitors. To cope with this limitation, we replicated Experiment 2 in the lab at Erasmus University Rotterdam, using International Psychology students who participated for course credit.

### Method

#### Participants

As the current experiment was run in the lab, we were constrained in the number of participants we could recruit (to a greater extent than on Mechanical Turk), and therefore we aimed to include 80 participants in the analysis. Ninety participants (23 male, mean age 20.02 years, range: 17–29 years) were recruited from the first year International Bachelor of Psychology students at the Erasmus University Rotterdam, where their English proficiency had to be sufficient, as determined by having a TOEFL grade above 80. Participants were tested in the lab, which is equipped with 22-in. TFT screens with a resolution of 1920 × 1200 and a ratio of 16:10.

#### Materials

The same materials as in Experiment 2a were used.

#### Design and procedure

The design and procedure were identical to Experiments 1 and 2a, except that participants were tested in the lab.

### Results and discussion

Ten participants were excluded from further analysis: three appeared to have reversed the keys and seven performed below the 80% accuracy cutoff. Like the other experiments, use of these exclusion criteria were preregistered on the OSF before data collection began. Eighty participants were included in the analysis. Furthermore, one item was removed from the analysis as an item analysis revealed an accuracy below our 80% cutoff. Fifteen experimental item pairs remained in the analysis.

A paired-samples *t* test was conducted to investigate whether there was a match advantage for accuracy and RTs using a stimulus set low in contrast, with saturation levels reduced to a point at which the hue was still recognizable. The results indicated no significant difference in accuracy scores between the match (*M* = .96, *SD* = .07) and mismatch conditions (*M* = .95, *SD* = .08), *t*(79) = 0.62, *p* = .534, *d* = 0.07. Participants produced faster responses in the match than in the mismatch condition (*M* = 846 ms, *SD* = 355 ms and *M* = 926 ms, *SD* = 548 ms, respectively), but this did not reach statistical significance, *t*(79) = −1.78, *p* = .080, *d* = 0.20, BF_10_ = 0.55. Comprehension accuracy was high (*M* = 0.82, *SD* = 0.14).

The results from Experiment 2b also support the results from Experiment 2a, as neither experiment found conclusive evidence for a match effect.

## Experiment 3

To further determine the effects of reduced saturation on the match advantage, Experiment 3 was run using the same pictures as Experiment 1 and 2, except they were shown in grayscale. As no hue is present in grayscale photos, no significant difference between the match and mismatch condition is expected.

### Method

#### Participants

Two hundred and twenty-two participants (98 males, mean age 38.64 years, range: 19–71 years) took part in the current study, and were recruited from Mechanical Turk and paid $1.50 for their participation.

#### Materials

The pictures used in this experiment were adapted from those used in Experiment 1 such that they were depicted in gray shades (see Fig. 1). The gray shades were achieved by changing the pictures to black and white by using Paint.NET software. The sentences remained unchanged.

#### Design and procedure

The design and procedure were identical to that of Experiments 1 and 2.

### Results and discussion

Forty-two participants were excluded from further analysis: Two reported that English was not their first language, seven appeared to have reversed the keys, 12 performed below the 80% accuracy cutoff, and 21 last-run participants were removed to equate the number of subjects per list. One hundred and eighty participants were included in the analysis.

A paired samples *t* test was conducted to investigate whether there was a match advantage for accuracy and RTs using pictures portrayed in grayscale. The results indicated that accuracy rates in the match condition (*M* = .97, *SD* = .06) and in the mismatch condition (*M* = .96, *SD* = .08) did not significantly differ, *t*(179) = 1.89, *p* = .06, *d* = 0.14. In the RTs there was also no significant difference between the match (*M* = 1,239 ms, *SD* = 641 ms) and mismatch conditions (*M* = 1,243 ms, *SD* = 558 ms), *t*(179) = 0.21, *p* = .834, *d* = 0.02, BF_10_ = 0.09. Comprehension accuracy was high (*M* = 0.81, *SD* = 0.19).

The results of Experiment 3 suggest that, when pictures are shown completely in grayscale, there is no significant match advantage present.

### Exploratory analyses

We were interested in examining exactly how the match and mismatch conditions differed from each other across experiments. As such, we conducted several exploratory analyses to gain a better appreciation of the processes that are occurring.

**Fig. 3:**
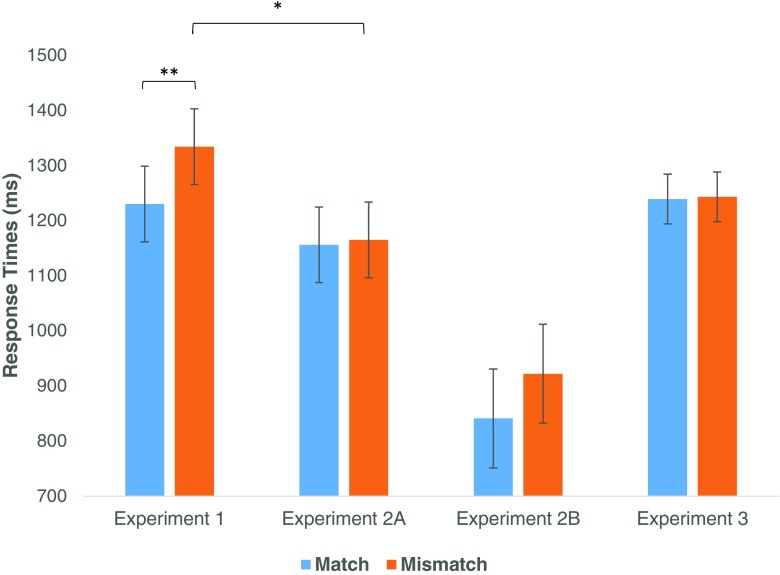
Size of match advantage in Experiment 1, when pictures were shown at normal levels of saturation; Experiments 2A and 2B, when saturation was reduced; and Experiment 3, when all pictures were shown in grayscale. **p < .01. *p < .05. (Color figure online)

We conducted a repeated-measures ANOVA over the reaction time data to examine the differences between Experiments 1, 2a, and 3, where “experiment” was the between-subjects factor, and we found that there was a significant main effect of condition, *F*(1, 453) = 5.01, *p* = .026, and a significant interaction between condition and experiment, *F*(2, 453) = 3.30, *p* = .038. No main effect of experiment was found, *F*(2, 453) = 1.58, *p* = .207. On the basis of these results we decided to run additional analyses to see whether the RTs from Experiment 2a significantly differed from Experiments 1 and 3 per condition. A simple contrast revealed that the RTs in the mismatch condition were significantly faster in Experiment 2a, *t*(274) = −2.26, *p* = .024, than in Experiment 1. No further significant interactions were found (see Fig. 3).

## General discussion

Previous research on the presence of color in mental simulations has come up with some contradictory findings (e.g., Connell, 2007; Zwaan & Pecher, 2012). One of the aims of the current study was to conclusively establish whether color is simulated or not. A second aim was to discover how much perceptual information is present in a mental simulation. Many object features have been studied in the past, but color is the only feature that can be decomposed while the object’s identifiability remains unchanged. It may be argued that the match advantage exists because if a picture matches the perceptual image in the mental simulation, then response time is facilitated. When there is a mismatch, this facilitation cannot occur and may instead result in interference, leading to longer response times.

In order to successfully complete our first aim, the experiments used more stringent criteria for the stimuli compared to what was used by Connell (2007) and Zwaan and Pecher (2012), as they included some items that could change shape as well as color. Furthermore, the median reaction time rather than the mean was used, such as in the replication by Zwaan and Pecher (2012) and other studies using a similar paradigm, as it allowed for less data to be discarded and was in line with the methods of previous research.

Experiment 1 found a significant match advantage of 104 ms, which supports the hypothesis that color is indeed present in mental simulations and supports the results by Zwaan and Pecher (2012) and Connell and Lynott (2009). In order to examine the richness of mental simulations and thus address our second goal, Experiment 2 used items where the saturation of the color was reduced to the lowest point at which the hue was still recognizable. The results of this experiment found no significant difference between the match and the mismatch condition. Interestingly, however, exploratory analyses revealed that the RTs in the mismatch condition were significantly faster in Experiment 2a than in Experiment 1, while no difference was found for the match condition. The results from Experiment 2a therefore serve to illustrate two points: First, the match advantage disappears when saturation in pictures is lowered, and second, the reason it disappears is due to a speeding up of response time in the mismatch condition. These results are intriguing as they suggest that, rather than a picture being *more* of a match leading to faster response times (i.e., facilitation), it would suggest that the match effect appears due to there being a *vivid* difference between the pictured object and the simulation in the mismatch condition, leading to interference effects. Experiment 3 provides tentative evidence in support of this hypothesis as well, as the average response times of this experiment appear to fall in between those of Experiment 1 and Experiment 2a, although this difference does not reach significance. As the average difference in reaction time between Experiment 2a and 3 is only 8 ms, it is unrealistic to expect a significant difference using a between-subjects analysis. It would be interesting for future studies to examine, using a within-subjects paradigm, at which level of saturation color can aid object recognition. Although the between-subjects comparison in our exploratory analyses were not significant, such future studies could illustrate that the mere presence of color—even at the lowest level of saturation during which the hue is still recognizable—serves to enhance performance in the object-verification task. Indeed, this is supported by the general literature stating that color aids in object recognition (Bramão et al., 2011).

In addition to finding a match advantage in the RTs of participants in Experiment 1, we also found a significant reduction in accuracy in the mismatch condition. As we removed items that had an average accuracy below 80%, this reduction cannot be explained by the pictures in that condition not matching the sentence. The match advantage in the RTs bear no relation to the accuracy scores, as only the RTs of accurate responses were used. This reduction in accuracy scores, however, could serve to explain why a match effect exists at all. We previously argued that the match effect exists due to a vivid difference occurring in the mismatch condition between the pictured object and the simulation. The task participants had to complete required them to only examine whether the actually pictured object (with no instructions mentioning color) was mentioned in the previous sentence. A strategy that could aid in the completion of such an object-verification task—in which speed is important—could be that participants simply judge whether the picture they see overlaps with what is present in the mental simulation. When there is a vivid difference, or no overlap, between the picture and the simulation, they are more likely to answer with an incorrect *no* response. It would be interesting to examine whether the removal of the instructions requiring speed would eliminate the difference in accuracy between the two conditions.

As for the “richness” of our mental simulations, we can conclude that they are rich indeed, in the sense that they include multiple object properties. We already know that color can be decomposed into different dimensions, namely hue, saturation, and brightness. If the reduction in the level of one of these dimensions (in our study: saturation) had not reduced the match advantage, we would have had to argue that color would not be present or relevant in a mental simulation. Our study, however, found that by reducing saturation, the match advantage disappears. Furthermore, we found tentative evidence that the mere presence of color—even with low levels of saturation—can aid in object recognition, compared to when color is removed entirely.

In sum, the current study found further support that color is another object property that is represented in mental simulations, in addition to shape and orientation. Furthermore, we have shown that by reducing saturation of the picture shown we can remove the match advantage as well, while still being involved in object recognition. This leads to the conclusion that, when comprehending language, we build mental simulations rich in perceptual detail.

## Footnotes

1 Amazon’s Mechanical Turk is an Internet marketplace that enables businesses/researchers to recruit participants for surveys/social science experiments.
